# Long-Range Spatial Distribution of Single Aluminum Sites in Zeolites

**DOI:** 10.1021/acs.jpclett.1c03554

**Published:** 2022-01-31

**Authors:** Enrico Salvadori, Edoardo Fusco, Mario Chiesa

**Affiliations:** Department of Chemistry and NIS Centre, University of Turin, Via Pietro Giuria 7, 10125 Turin, Italy

## Abstract

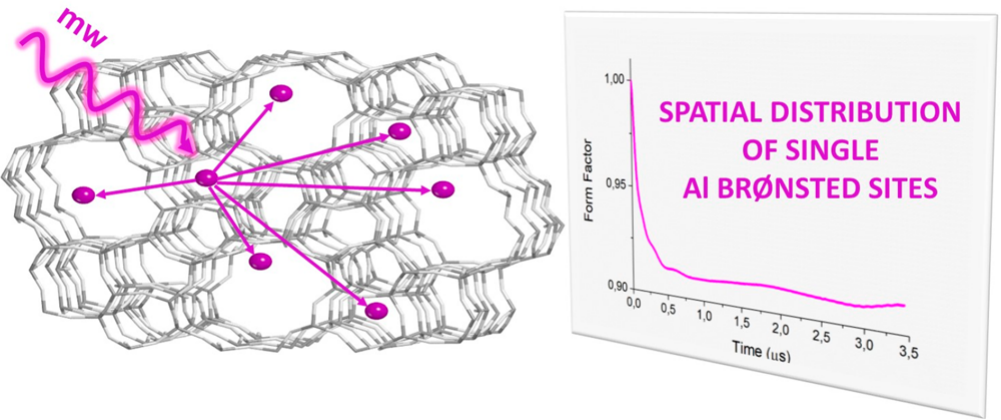

How aluminum distributes during synthesis and rearranges after processing within the zeolite framework is a central question in heterogeneous catalysis, as it determines the structure and location of the catalytically active sites of the one of the most important classes of industrial catalysts. Here, exploiting the dipolar interaction between paramagnetic metal ions, we derive the spatial distribution of single aluminum sites within the ZSM-5 zeolite framework in the nanometer range, in polycrystalline samples lacking long-range order. We use a Monte Carlo approach to validate the findings on a pristine ZSM-5 sample and demonstrate that the method is sensitive enough to monitor aluminum redistribution induced in the framework by chemical stress.

The chemistry and catalysis of zeolites are driven by the presence of aluminum ions in tetrahedral coordination at so-called crystallographic T sites, which, for charge compensation, introduce Brønsted (H^+^) or redox (transition metal cations) functionalities that serve as catalytic active sites.^1,2^ To harness the catalytic potential of such sites and implement catalyst performances by design, a precise description of the Al site distribution is required. In particular, knowledge of the (i) Si/Al ratio; (ii) the distribution of the Al atoms among crystallographic unique lattice sites; and (iii) the relative proximity of lattice Al atoms (i.e., the distribution of interatomic Al–Al distances among framework Al-site pairs) is crucially needed. Experimentally, the last two points are particularly taxing to achieve.^3,4^ In fact, although the spatial density of Al atoms is decisive in determining the turnover rate of a catalytic reaction, its experimental assessment remains an open challenge. In the literature, there are several types of evidence pointing toward a nonrandom distribution of aluminum within the zeolite,^4,5^ but very few experimental methods are available to directly determine the long-range spatial distribution of such sites in disordered polycrystalline materials. Here, we address this for the ZSM-5 zeolite, a particularly interesting case for this topic in light of its high industrial interest^6^ and its inherent topological complexity.^7^

ZSM-5 is commercially one of the most widely used Si-rich (Si/Al > 12) zeolites. It has an MFI structure characterized by the intersection of straight and sinusoidal 10-membered ring channels (of approximately 5.5 Å in diameter) and larger spherical voids at the channel junctions (of ∼10 Å in diameter). The MFI topology comprises either a monoclinic (*P*21*/n* symmetry) or orthorhombic structure (*Pnma* symmetry) with 24 and 12 crystallographically distinct T sites, respectively, the 24 T sites of the monoclinic form corresponding to the 12 T sites of the orthorhombic form.^8^ The T1, T2, T3, T5, T6, T7, T9, and T12 sites are accessible within channel intersections, while T4 and T10 are within the sinusoidal channel, and T8 and T11 are within the straight channel.

The high Si/Al ratios and the very similar scattering factors for Al and Si make the determination of the Al atoms at the different T sites of ZSM-5 by X-ray diffraction (XRD) very difficult. To circumvent the problem, Seff and co-workers^9,10^ exchanged the protons of H-ZSM-5 with the much heavier thallium or cesium cations (i.e., Tl-ZSM-5 and Cs-ZSM-5) providing evidence by XRD that the distribution of Al over the T sites is not random. The same indications have been obtained by energy dispersive X-ray spectroscopy (EDX)^11^ and atomic probe tomography (APT).^12^

^27^Al NMR techniques,^13−15^ often assisted by DFT modeling,^16^ have proven very useful in assessing the distribution of Al species in zeolite catalysts at atomic length scales, providing evidence for the nonrandom nature of the Al site distribution suggesting that the number of distinct Al sites could be as low as 3 over 24 possible substitution sites. More recently, the preferential incorporation of Al at distinct tetrahedral sites of as-synthesized ZSM-5 (containing the organic structure directing agent) has been proposed based on a combination of DFT calculations and two-dimensional ^29^Si–^27^Al NMR experiments,^17^ while by means of ^27^Al MQMAS NMR at 22.3 T the distribution of aluminum over the tetrahedral sites and their evolution after steam treatment has been assessed.^15^ The general conclusion from these studies is that the Al siting is neither random nor controlled by a simple rule but depends on synthetic protocols and postsynthesis treatments.^16^ In this context, while the combination of specific spectroscopic techniques and DFT modeling can provide structural insights at the atomic length scale, the determination of the long-range spatial distribution of Al sites in Si-rich zeolites remains an open challenge of particular interest.

To address this issue, in this contribution, we selectively labeled isolated aluminum sites in ZSM-5 zeolite (Figure 1a,b) with paramagnetic Zn^I^ ([Ar]3d^10^4s^1^, *S* = 1/2)^18,19^ and exploited the long-range dipolar interaction between electron spins to measure their distribution within the zeolite framework.

**Figure 1:**
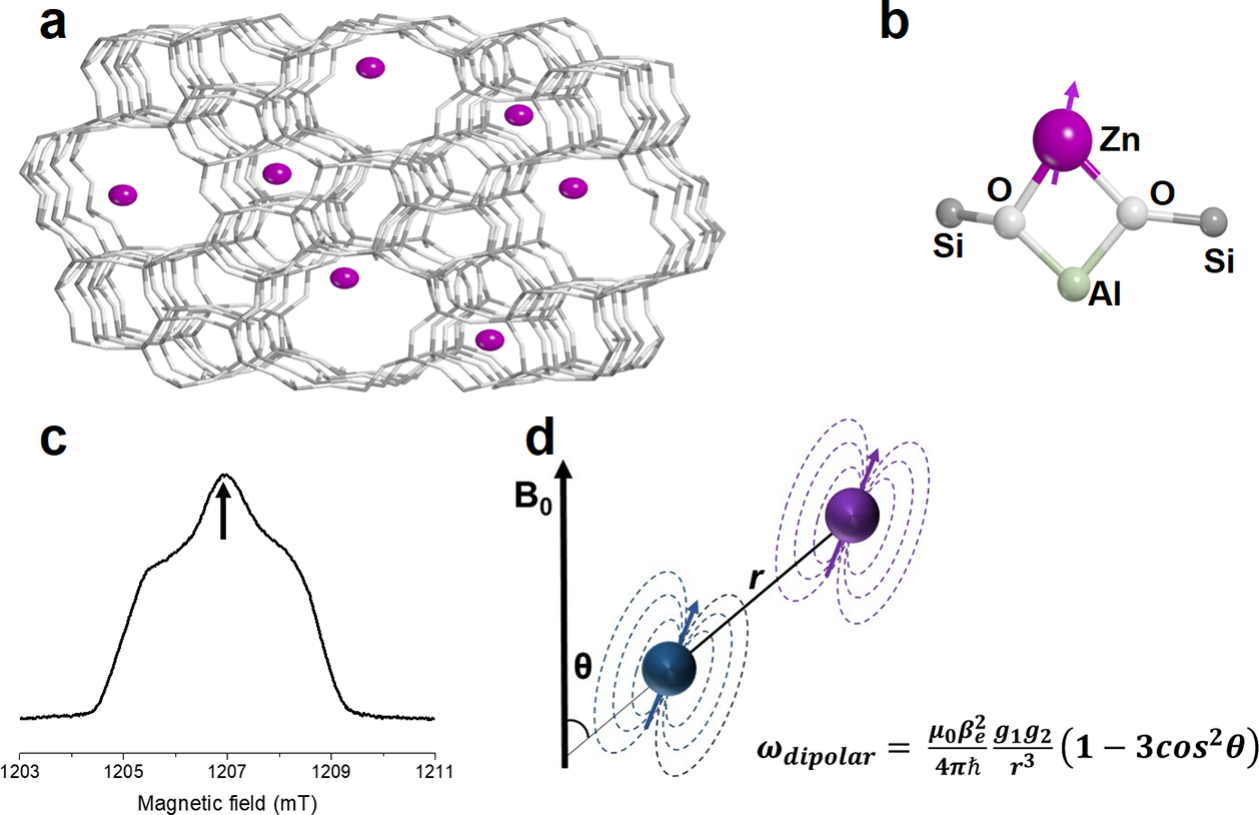
Overview of the Zn^I^-loaded ZSM-5 structure and the physical principle of magnetic dipole–dipole interaction. (a) Pictorial rendering of the Zn^I^-loaded ZSM-5 sample. The zeolite framework is reported in white (oxygen) and gray (silicon), while Zn ions are depicted as purple spheres. (b) Close-up on the geometrical structure of the Zn^I^ site as derived by EPR spectroscopy data.^18,19^ The purple arrow represents the electron spin. Color code: purple: zinc; white: oxygen; gray: silicon; sage: aluminum. (c) Room-temperature Q-band (33.7658 GHz) echo-detected EPR spectrum of Zn^I^; see also Figure S2. The arrow marks the field position used to record the RIDME time trace. (d) Schematic representation of the magnetic dipole-magnetic dipole interaction between two electron spins in the presence of an external magnetic field. The interspin vector, *r*, makes an angle θ with the external applied magnetic field, *B*_0_.

The formation of monovalent Zn cations (Zn^I^) results from the spontaneous ionization of Zn atoms only at isolated Al–OH sites (Figure 1c,d and Figures S1 and S2), as demonstrated by EPR studies.^18,19^ Zn^I^ features some intriguing properties that emerge from its geometric and electronic structure, namely, long spin relaxation times even at room temperature (*T*_m_ ≈ 2 μs and *T*_1_ ≈ 16 μs) and a rigid coordination geometry (Figure 1b). These make Zn^I^ an ideal candidate as a fully inorganic spin label.

Indeed, the magnetic electron–electron dipole interaction between pairs of Zn^I^ ions enables the determination of their spatial density distribution in the nanometer range by pulse dipolar spectroscopy (PDS); see Figure 1d and Figure S3. The PDS data are recorded as modulation that depends on the Zn^I^–Zn^I^, hence aluminum–aluminum, distance distribution which can be derived with high resolution.^20,21^ In the point dipole approximation, the dipolar frequency can be written as , where *g*_1_ and *g*_2_ are the characteristic isotropic *g*-values for the two spin species (in the present case *g*_1,2_ = 1.998, the average *g*-value of Zn^I^), and β_e_ is the Bohr magneton. As an example assuming a random distribution (spherical average), interspin distances of **2** and **5** nm translate to dipolar frequencies of 6.4771 and 0.4145 MHz, respectively. With a magnetic dipole moment 660 times larger than that of a proton (∼−9.285 × 10^–24^ JT^–1^ as opposed to ∼1.411 × 10^–26^ JT^–1^), an electron spin gives access to distances up to ∼15 nm, which are much longer than those accessible through NMR.

When, as in the present case, multiple or distributed distances are present, each contributes individually to the measured time trace so that PDS may yield not only an average distance(s) but also their relative distribution. Such an approach has found widespread use in structural biology.^22,23^ However, at variance with spin-labeled biological systems where a sample is constituted by identical copies, spin labeling in polycrystalline zeolites has a degree of variability dependent on silicon-to-aluminum and aluminum-to-spin label ratios. To take this into account, we developed tailored methods to interpret the data based on a Monte Carlo approach.

Among the available PDS sequences,^20,21^ for this work we selected the Relaxation Induced Dipolar Modulation Enhancement (RIDME) pulse sequence which exploits spontaneous relaxation events to retrieve the dipolar coupling;^24^ see Figure 1d and Figure S4. Figure 2a reports the room-temperature RIDME data after removal of the background decay (form factor), which shows a pronounced damping of the dipolar oscillations indicative of multiple or distributed distances. As a first approximation, to obtain the relative distance distribution, the RIDME trace was analyzed with DeerAnalysis,^25^ which employs a model-free Tikhonov regularization algorithm to derive a distance distribution without any prior knowledge or assumptions (hereafter “model-free distribution”). As shown in Figure 2b, the model-free distance distribution is not uniform over the entire range, as it would be expected if no preferential sites were populated. Rather, it displays maxima and minima compatible with specific siting.

**Figure 2:**
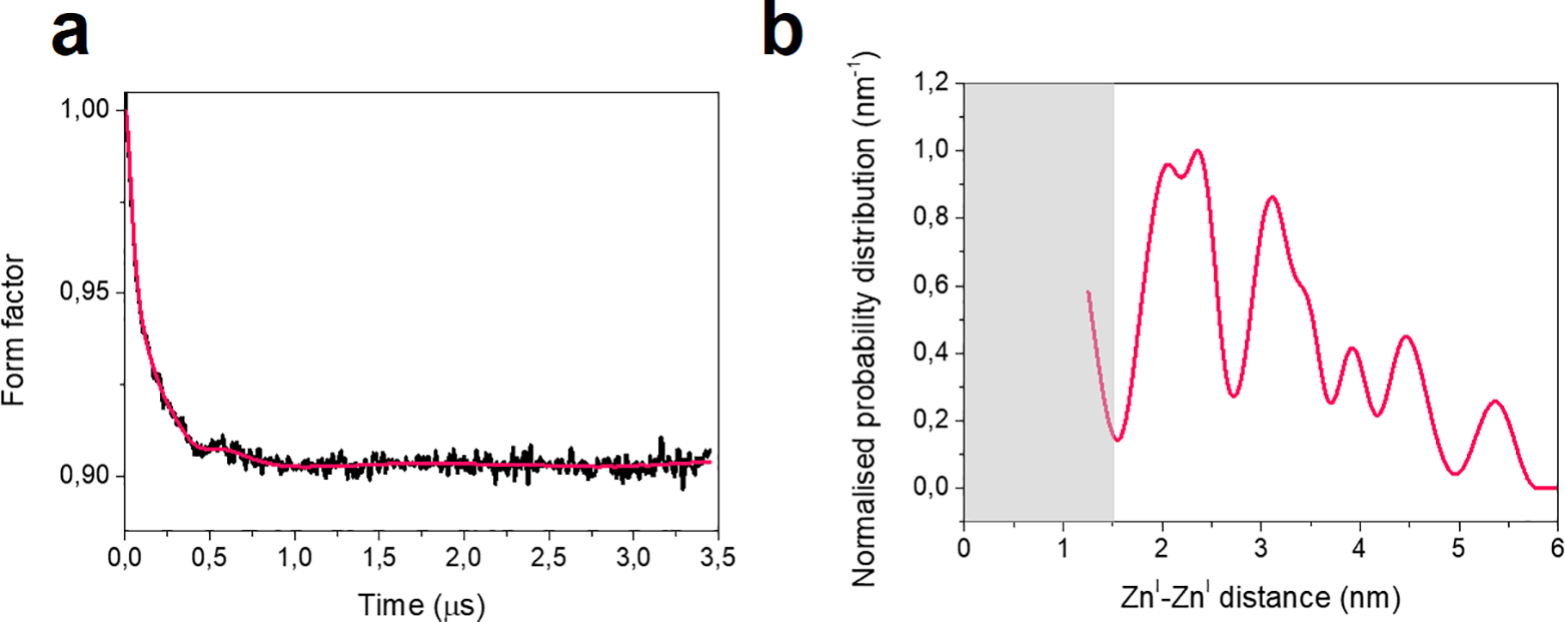
Room-temperature RIDME experimental data and extracted distance distribution. (a) Background-divided time trace (black) with corresponding fit (red) based on the model-free analysis (DeerAnalysis2018). The primary data are reported in Figure S5, whereas data on independent sample preparations are reported in Figure S6. (b) Model-free distance distributions corresponding to the fitting in a obtained using Tikhonov regularization in the DeerAnalysis toolbox. The region shaded in gray represent the 1.5 nm cutoff necessary to employ the point dipole approximation. The experimental trace was recorded at room temperature.

To assess the origin of such a discrete distribution and whether it allows the discrimination of specific siting sites among the 24 potentially available (named T1–T24), we turned to a Monte Carlo approach. This step is necessary since in a sample no two crystallites are exact copies. On the basis of the crystal structure for the dehydrated H-ZSM5 at room temperature, we computed the expected Zn^I^···Zn^I^ distances considering that each Zn^I^ randomly occupies either a single site (T7, T8, and T10) or two sites (T7 and T10). These sites have been selected based on previous diffraction and spectroscopic studies.^9,10,26^ Moreover, as a point of reference, the expected Zn^I^···Zn^I^ distances for a completely random distribution (all 24 sites populated with equal probability) were evaluated (All Ts). The result of this analysis is presented in Figure 3a as histograms. These distance probability distributions were used to compute the corresponding form factors considering the distances between 1.6 and 5 nm. The lower limit complies with the Löwenstein rule^27^ and the point dipole approximation for the magnetic dipolar coupling, whereas the upper limit is consistent with the length of the experimental trace. In zeolites, distances <1.6 nm are in principle possible but are not suitable for PDS because the dipolar frequency would be contaminated by the exchange interaction. Such distances are best assessed by considering the line broadening of the EPR line. However, such an effect was found negligible on these samples. The computed form factors were then analyzed in the same way as the experimental data to yield the corresponding model-free distance distribution. Figure 3b reports a comparison between the calculated (T7) and experimental form factors, while Figure 3c compares the model-free distance distributions and shows that the distribution broadens and becomes progressively less defined the more sites are considered.

**Figure 3:**
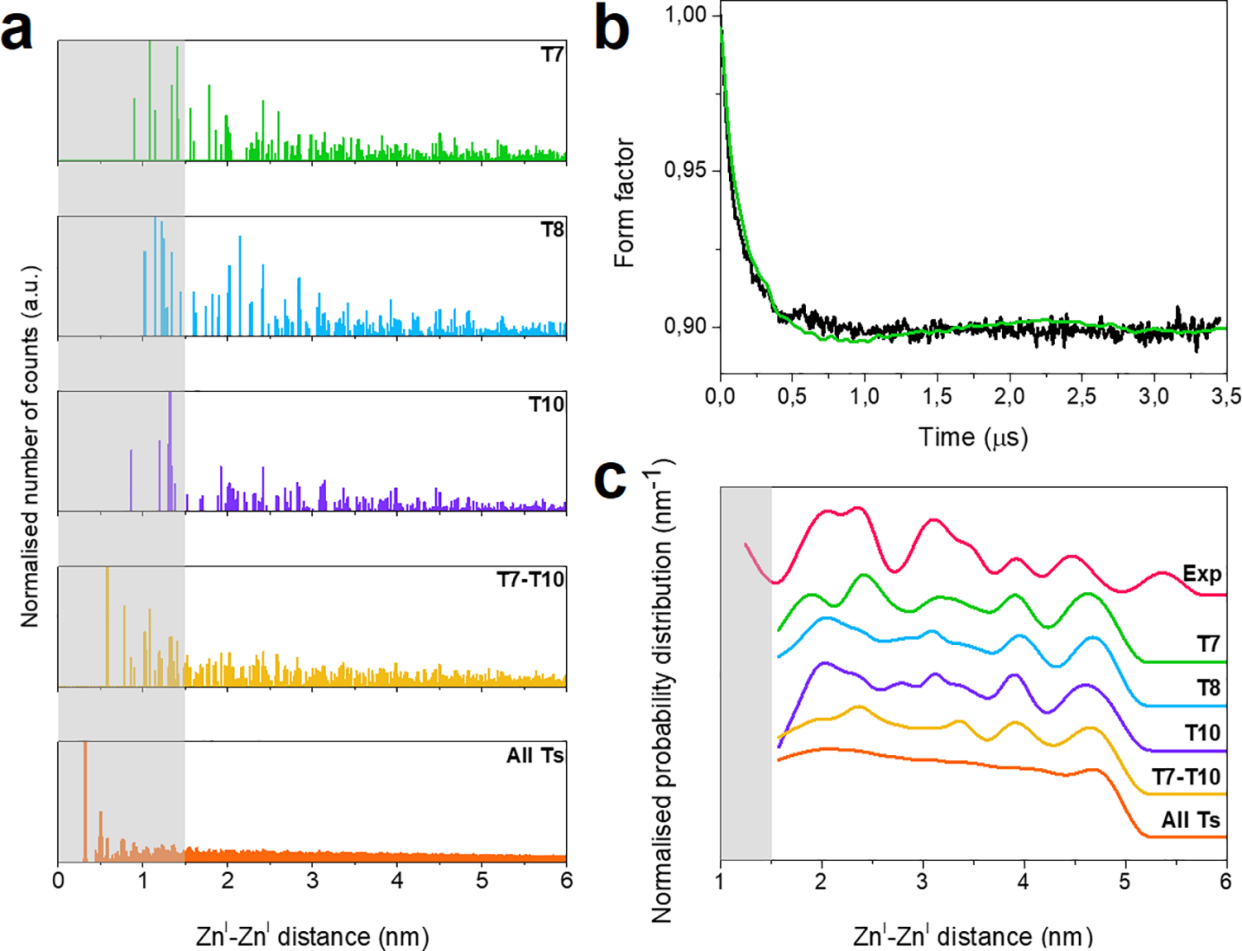
Comparison between the Monte Carlo statistics and experimental data. (a) Histograms reporting the normalized distribution of the Zn^I^···Zn^I^ distances in the range 0–6 nm as computed through the Monte Carlo approach for the individual sites T7, T8, T10, for the pair of sites T7–T10, and for all T sites. The bin size is 0.2 Å. (b) Overlay of the calculated form factor for the site T7 (green line) and the experimental form factor (black line) measured at room temperature. The calculated form factor is based on the distance distribution reported in (a) considering all distances between 1.5 and 5 nm. The apparent noise in the calculated form factor is the result of the discrete number of counts. (c) Calculated distance distributions obtained using Tikhonov regularization for the sites T7, T8, T10, the pair of sites T7–T10, and all T sites compared with the experimental result (red line). For better visualization, the distance distributions have been vertically offset. The calculated form factor is based on the distance distribution reported in (a) considering all distances between 1.5 and 5 nm. The region shaded in gray represents the 1.5 nm cutoff necessary to employ the point dipole approximation.

This comparison provides evidence that Zn^I^ ions, and therefore aluminum ions, do not occupy random but preferred sites within the ZSM-5 structure and that PDS is sensitive enough to pinpoint such specific sites. In fact, the distance distributions computed through the Monte Carlo approach show distinct patterns that, in principle, allow one to distinguish between T sites; see Supporting Figure 7 for an extended comparison. Note that, as a consequence of the crystalline nature of the zeolite framework, the clearest differences manifest at short distances (<3 nm) and effectively provide a unique “barcode” to identify each T site. At longer distances, all distributions display a similar pattern due to the average crystal structure rather than single-site behavior. Even restricting to distances <2 nm, only sites T1, T4, T7, and T11 show patterns compatible with the experimental results. Of these, only T1^17^ and T7^9,10,19^ have been already implicated as probable aluminum substitution sites. Considering the replicas reported in Supporting Figure 6, we estimate that the uncertainty in the position of the maxima of the distance distribution (below 3 nm) is on the order of ±0.25 nm.

Having established that PDS provides meaningful data even in the case of complex, disordered samples such as ZSM5 zeolites, we set out to examine whether PDS is sensitive enough to detect how the aluminum probability distance distribution varies upon chemical treatment. To this end, we selected a steaming procedure that is known to cause a rearrangement of the Al sites and a redistribution of Brønsted and Lewis acid sites.^28^Figure 4 reports a comparison of the experimental form factor before and after steaming and the relative model-free distance distributions. As is readily apparent, the distance distribution varies considerably in the range 1.5–2.7 nm before and after treatment, with the appearance of two discrete peaks at 1.91 and 2.48 nm for the steamed sample. Remarkably, all other distances remain unchanged highlighting that the structural modification is mostly short-range, at least as the active sites capable of stabilizing Zn^I^ are concerned. These results are in line with atom probe tomography data, which revealed a nonrandomness for the Al distribution even for highly crystalline materials and reported a most probable Al–Al neighbor distance of 1.80 ± 0.6 nm for a pristine ZSM-5 zeolite (Si/Al = 17) and of 0.9 ± 0.3 nm for the same ZSM-5 zeolite after severe steaming.^12^

**Figure 4:**
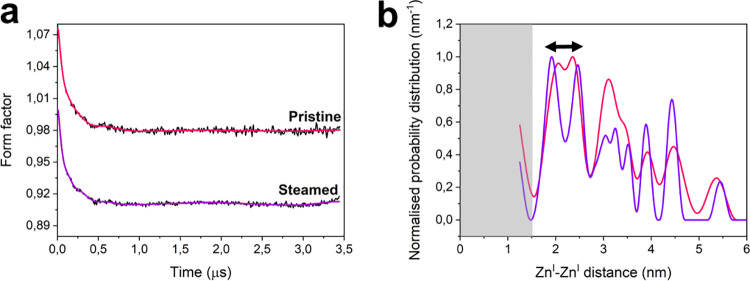
Effect of chemical stress (steaming) on the measured distance distribution. (a) Background-divided time traces (black) with corresponding fits (red and purple) based on the model-free analysis. For better visualization, the RIDME time traces have been vertically offset. Both traces were recorded at room temperature. The primary data and the validation analysis are reported in Figure S5. (b) Distance distributions corresponding to the fitting in (a) obtained using Tikhonov regularization in the DeerAnalysis toolbox. The double-headed arrow marks the shift in the range 1.91–2.48 nm after steaming. The region shaded in gray represents the 1.5 nm cutoff necessary to employ the point dipole approximation.

In summary, we proved that PDS is an effective and accurate method to derive distances in the nanometer range for disordered polycrystalline inorganic materials and that Zn^I^ is a convenient spin label for the determination of the isomorphous aluminum distribution within the zeolite framework. We showed that the dipolar time trace and relative distance distribution are incompatible with a nonpreferential population of all T sites, but rather that Zn^I^—and hence aluminum—preferentially binds to a restricted number of T sites and that PDS is sensitive enough to capture the redistribution of aluminum after chemical treatment. To the best of our knowledge, this is the first method able to give a nanometer-range distribution of active sites in complex solid-state systems such as zeolites. As a word of caution, we note that the measured distance distribution depends on the sample and on the paramagnetic probe employed, as different probes may prefer different anchoring sites, and the distribution of aluminum Brønsted sites is influenced by the silicon-to-aluminum ratio and the synthetic protocol used. Moreover, paramagnetic labeling of polycrystalline inorganic materials invariably shows some degree of variability; this is because—at odds with site-direct spin-labeling in biological samples—multiple anchoring sites are present, and it is not possible to exert fine control over the degree of occupancy. This is reflected in the variability of the associated distance distributions, which, far from constituting a limit, is a reporter of the intrinsic complexity of such systems. We believe that PDS may become a powerful tool in the characterization of inorganic systems especially in combination with local probe techniques (e.g., NMR). We also anticipate that the approach could be extended to other paramagnetic species on polycrystalline inorganic supports lacking long-range order to probe their spatial density and proximity, a crucial factor to tune catalytic pathways and turnover rates.^3^

## Materials and Methods

### Sample Preparation

#### Zeolite Pretreatment

The H-ZSM-5 zeolite (Si/Al = 40, supplied by Haldor Topsøe) was dehydrated by thermal treatment at 393 K under a dynamic vacuum (residual pressure < 10^–4^ mbar) for 2 h and calcinated at 773 K in an O_2_ atmosphere (390 mbar) to remove spurious organic residues. Excess O_2_ was subsequently removed by a dynamic vacuum (residual pressure <10^–4^ mbar).

#### Zeolite Steaming

The calcinated H-ZSM-5 zeolite was exposed to 50 mbar of water vapor at room temperature before being heated to 973 K (20 K min^–1^ ramp) for 0.5 h. Subsequently, the sample was allowed to slowly cool to room temperature in the oven. The cycle was repeated three times, and between each cycle the sample was left at 373 K for 1 h. Lastly, the sample was dehydrated by thermal treatment at 673 K under a dynamic vacuum (residual pressure < 10^–4^ mbar).

#### Zn Evaporation/Illumination

The Zn/ZSM-5 samples were prepared by sublimation of metallic zinc on the protonated ZSM-5 zeolite either directly after pretreatment or after steaming. The activated zeolite was exposed for 2 min to metallic zinc vapors generated in situ by heating a zinc metal bead (approximately 1 mm in diameter) at 673 K. The Zn vapor pressure at this temperature was 0.4 mbar. All samples were sealed under a vacuum and proved to be stable for several months.

The formation of Zn^I^ strictly requires the presence of a single Brønsted site. The formation of oxidized Zn species by the reaction of metallic Zn with surface silanols or extra-framework aluminum cannot be ruled out; however, these are expected to be diamagnetic Zn^II^ species that are EPR silent. XANES data reported on the same system in ref (18) show that metallic Zn and ZnO particles, if at all present, are not formed in appreciable yield.

#### Zn/Al Quantification

Total content determination of Al, Si, and Zn were performed by inductively coupled plasma–optical emission spectroscopy (ICP-OES – Optima 7000 DV PerkinElmer) equipped with a Cross Flow nebulizer, a Scott spray chamber, and an Echelle monochromator. The wavelengths employed for the quantification were 396.153 nm (Al), 212.412 nm (Si), and 206.412 nm (Zn). The dissolution procedure consisted of an acid digestion in a microwave oven (milestone MLS-1200 MEGA). Twenty milligram sample aliquots were treated with a mixture of 5 mL of aqua regia and 2 mL of hydrofluoridic acid in tetrafluoromethoxyl (TMF) bombs. Four 5 min heating steps (250, 400, 600, 250 W microwave power, respectively) were each followed by a 25 min ventilation step. Subsequently, 0.7 g of boric acid were added, and the bombs were further heated at 250 W for 5 min and then cooled by a ventilation step of 15 min. At the end of the treatment, the samples appeared fully dissolved. Finally, the resulting solutions were diluted to 25 mL with HPW. Each sample was analyzed in duplicate, and each reported concentration was averaged on the basis of three instrumental measurements. ICP analysis yielded a Si/Al content in line with the nominal value and a Zn/Al content on the order of 1.00–1.40.

### EPR Spectroscopy

#### Spectrometer Description

Q-band pulse EPR experiments were performed at 298 K on a Bruker ELEXYS 580 EPR spectrometer (microwave frequency ≈ 33.7 GHz) equipped with a Bruker EN 5107D2 resonator and an Oxford Instruments CF935 liquid-helium cryostat. The magnetic field was measured by means of a Bruker ER035 M NMR gaussmeter. All EPR measurements were performed at room temperature.

#### Echo-Detected Field Sweep (EDFS) EPR Spectrum

The EDFS EPR spectrum was recorded with the standard Hahn echo sequence π/2−τ–π-echo at 33.7658 GHz at room temperature with π/2 = 16 ns, π = 32 ns, and τ = 200 ns.

#### Determination of Relaxation Times

*(T*_*m*_*and T_1_)*. The phase memory time (*T*_m_) was measured using the standard two-pulse echo sequence π/2−τ–π–τ–echo where the interpulse delay τ had an initial value of 200 ns and was incremented in steps of 16 ns. The longitudinal relaxation time (*T*_1_) was measured with the standard inversion recovery pulse sequence π–T−π/2−τ–π–τ–echo; the time delay *T* had an initial value of 1000 ns and was varied in steps of 500 ns. In both sets of measurements, *t*_π/2_ = 16 ns and *t*_π_ = 32 ns. The shot repetition rate was 0.5 and 2 kHz for *T*_m_ and T_1_, respectively. Both *T*_m_ and T_1_ time traces were fitted with a stretch exponential decay function, yielding for the pristine ZSM-5 *T*_1_ = 15.644 μs (0.94), *T*_m_ = 1.721 μs (0.73), and *T*_1_/*T*_m_ = 9.09 and for the steamed ZSM-5 T_1_ = 15.897 μs (0.95), *T*_m_ = 1.894 μs (0.80), and *T*_1_/*T*_m_ = 8.39. The numbers in parentheses represent the stretching factors. A relative long *T*_m_ is needed for sensitivity and to access long distances (up to ∼6 nm), while a short *T*_1_ is desirable for fast signal averaging. A ratio 3 < *T*_1_/*T*_m_ < 10 was determined desirable for RIDME sensitivity.

#### RIDME Pulse Sequence

The dead time free, five-pulse RIDME introduced by Milikisyants et al.^24^ sequence was used: *π/2*-τ_1_-π–τ_2_-*π/2*-*t*_mix_-*π/2*-τ_3_-π–τ_4_-echo, also shown in Figure S4. The experimental values were as follows: *π/2* = 16 ns, π = 32 ns, τ_1_ = 400 ns. The initial position of the mixing block (*π/2*-*t*_mix_-*π/2*) was 140 ns before the primary echo, and the length of the RIMDE time trace was 4000 ns. The delay τ_2_ was incremented in steps of 16 ns, while τ_3_ was decremented by the same amount. In order to try and optimize the sensitivity, experiments were conducted for three values of *t*_mix_, namely, 12, 16, and 20 μs. Since no significant improvement was found for longer *t*_mix_, 12 μs was chosen as the optimal value. In order to reduce nuclear modulation (ESSEM) effects due to ^27^Al, a series of experiments employing a suppression cycle was conducted in which τ_1_ was incremented 8 times in steps of 22 or 100 ns;^29^ however, no visible difference was observed. An eight-step phase cycle was applied to remove phase offsets and echo crossing effects. The shot repetition time was 500 μs. Each time trace consists of 250 points. The background decay was fitted to a stretched exponential function *I*(*t*) = *e*^–*kt*^*d*/3^^, with dimensionality 4.7791 and 3.6468 for the pristine and steamed samples, respectively. This is common for RIDME experiments to account for spectra diffusion processes.^30,31^

#### 4p-DEER (PELDOR) Pulse Sequence

DEER (PELDOR) experiments were performed at room temperature on a Bruker ELEXSYS E580 spectrometer operating at 33.846 GHz equipped with a Bruker EN 5107D2 resonator housed in an Oxford Instruments continuous flow cryostat (CF935), at the University of Padova (Italy). The four-pulse DEER sequence used was *π/*2(ν_obs_)−τ_*l*_–π(ν_obs_)–*t′*–π(ν_pump_)–(τ_*l*_ + τ_2_–*t*′)−π(ν_obs_)−τ_2_–echo, where the observer pulse length was 24 ns for π/2 and 48 ns for π pulses; see Supporting Figure 9. The pump pulse length was also 48 ns. The first interpulse delay (τ_*l*_) was 300 ns, whereas the long interpulse delay (τ_2_) was 2500 ns. The pump and observer pulses were set on the high-field and low-field shoulder of the EPR spectrum, respectively. The background was corrected by a homogeneous three-dimensional exponential, and the distance distributions were evaluated by either Tikhonov regularization; see Supporting Figure 10.

### Data Analysis and Theoretical Prediction of Zn–Zn Distances

#### Model-Free Analysis

As a starting point, data were analyzed using the DeerAnalysis^25^ program developed by Gunnar Jeschke. This program allows the computation of a model-free distance distribution. The sample is considered as a collection of nanoobjects, each containing one or two electron spins. The advantage of DeerAnalysis is that it has been extensively tested by the EPR community on a large number of real (mostly biological) samples and is therefore very reliable. The best witting was chosen on the basis of the L curve criterion.

#### Monte Carlo Analysis

As opposed to biological samples where site-directed mutagenesis allows one to precisely and reproducibly introduce a spin label at specific sites, generating identical copies of the same protein, the ZSM-5 sample is constituted by a large number of particles, in which the Zn^I^ ion occupies randomly the available lattice positions. Therefore, to analyze the experimental data, we apply a Monte Carlo approach. As a starting point, we used the X-ray CIF file “ZSM-5, Calcinated” downloaded from the Database of Zeolite Structures.

We considered a ZSM-5 particle that comprises 4 by 4 by 4 elementary unit cells. This cubic particle has a maximum dimension, along the diagonal, of ∼25 nm and is therefore significantly larger than the largest distance detectable with the experiment, ∼7 nm. We then isolate the spatial coordinates of the aluminum sites of interest to generate individual data sets for single sites (T1 through T24), pairs of sites (e.g., T7–T10), as well as a single data set containing all sites (all Ts). In this approach, the Zn^I^···Zn^I^ distances are considered as the Si···Si distances. Each data set is treated in the same way to generate a probability distribution and the corresponding dipolar form factor. Namely, of all sites available within a data set, we randomly selected a subset which represents the Zn^I^ occupancy. For the selected sites, we computed the Euclidean distance between all possible pairs and used these distances to generate a probability histogram (with an edge resolution of 0.2 Å = 0.02 nm). In order to account for the random distribution of Zn^I^ sites, the procedure was repeated a large number of times (20 000) to generate replicas of the system and all probability distributions summed together to generate a global distribution. Because of the low occupancy, large distances are overrepresented, and therefore the computed distance distribution is normalized to the volume.

The form factor is computed by multiplying the relevant dipolar trace by the number of counts determined through the Monte Carlo approach and summing up the individual dipolar traces. This last approximation holds as the low occupancy and randomness in Zn^I^ distribution allow one to consider the experimental time trace as the sum of pairwise dipolar oscillations, not as the product of all possible dipolar traces.

Data were analyzed and processed with a home-written MATLAB (The MathWorks Inc., Natick, MA, USA) script. See also Supporting Figures 11 and 12.

## Supporting Information Available

Spectroscopic properties of Zn^I^, dependence of the dipolar frequency on the interspin distance, details on the RIDME pulse sequence, RIDME primary data and repeats, RIDME data on independent sample preparations, Monte Carlo computations on all T sites, the ESEEM effect, DEER data, independent sites approximation, effect of Zn^I^ occupancy (PDF)

## Notes

The data are available from the corresponding author upon reasonable request.

## Dedication

E.S. wishes to dedicate this paper to Bruna Pesce and Giovanni Sovilla.
