# Supplementary material for: Long-Range Spatial Distribution of Single Aluminum Sites in Zeolites
Source: J Phys Chem Lett. 2022 Jan 31;13(5):1283–9. doi: 10.1021/acs.jpclett.1c03554 (PMC8842299; doi:10.1021/acs.jpclett.1c03554)
Supplement: Supplementary file 1 — jz1c03554_si_001.pdf [file jz1c03554_si_001.pdf]

Supporting information for

Long-range spatial distribution of single aluminium sites in zeolites

Enrico Salvadori[1*], Edoardo Fusco[1†], Mario Chiesa[1]

[1]Department of Chemistry and NIS centre, University of Turin, Via Pietro Giuria 7, 10125 Turin, Italy

[†]Current address: School of Chemistry and EaStChem, University of St Andrews, St Andrews, KY16 9ST, United Kingdom

Corresponding author: enrico.salvadori@unito.it

Contents

THE PARAMAGNTIC Zn^{I} ION S2
DEPENDECE OF THE DIPOLAR FREQUENCY ON THE INTERSPIN DISTANCE S3
THE RIDME PULSE SEQUENCE S4
RIDME PRIMARY DATA S5
RIDME DATA ON INDEPENDENT SAMPLE PREPARATIONS S6
MONTE CARLO COMPUTATIONS ON ALL T SITES S7
THE ESEEM EFFECT S8
DEER SPECTROSCOPY S9
THE INDEPENDENT SITES APPROXIMATION S10
EFFECT OF THE Zn^{I} OCCUPANCY S11
REFERENCES S12

THE PARAMAGNTIC Zn^I ION

Sublimation of metallic Zn on thermally dehydrated H-ZSM-5 zeolite lead to the appearance of a distinct CW-EPR signal centered at about g = 1.998, which is assigned to Zn^I species with a $^2S_{1/2}$ ground state [1]. Analysis of the ^{67}Zn and ^{17}O hyperfine couplings demonstrated that the spin is strongly localized on the Zn nucleus (77%) [2]. The chemical reaction leading to the formation of Zn^I is pictorially represented in Fig. S1.

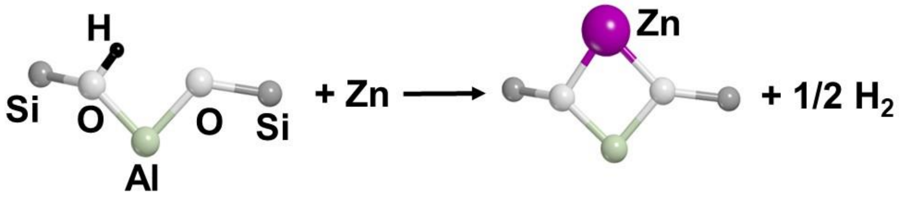

Fig. S1: Pictorial representation of the Zn^I insertion in ZSM-5. Zn^I ions are generated by directly sublimating metallic Zn on dehydrated acid H-ZSM-5.

Q-band (~ 33.7 GHz) CW EPR allows to resolve the full g-anisotropy and hyperfine interaction with ^{27}Al, see Fig. S2. For comparison Fig. S2 also reports the corresponding Q-band echo-detected field sweep spectrum.

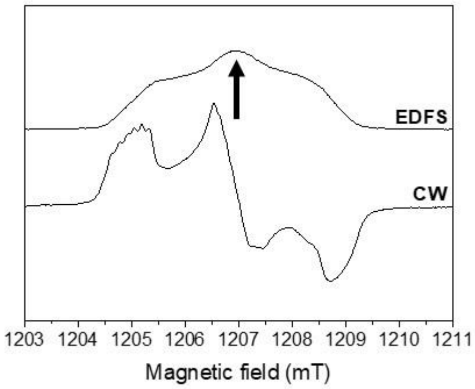

Fig. S2: The room-temperature Q-band field-swept Hahn echo-detected EPR spectrum (EDFS) of complex Zn^I loaded ZSM-5 and the corresponding room-temperature continuous-wave (CW) EPR spectrum. The EDFS spectrum was recorded at 33.7658 GHz, using the Hahn echo sequence with $\pi/2$ = 16 ns, π = 32 ns and τ = 200 ns. The CW spectrum was recorded with the following experimental parameters: microwave frequency = 33.73754 GHz, microwave power = 1 mW, modulation amplitude = 0.1 mT, modulation frequency = 50 kHz, conversion time = 81.92 ms, time constant = 40.96 ms. The arrows indicate the resonance field positions used in the RIDME experiments. The narrow spectral width of the EPR spectrum precludes the use of two-frequency PDS pulse sequences, such as DEER/PELDOR. All spectra were recorded at room-temperature. The relevant parameters derived from the EPR spectra are g = [1.9951 1.9984 2.0015] and A^{27Al} = [2.7 2.8 4.6] MHz [1]. All spectra were recorded at room temperature.

DEPENDECE OF THE DIPOLAR FREQUENCY ON THE INTERSPIN DISTANCE

In the point dipole approximation the dipolar frequency can be written as $\omega_{dipolar} = \frac{\mu_0 \beta_e^2}{4\pi\hbar} \frac{g_1 g_2}{r^3}(1 - 3cos^2\theta)$, where g_1 and g_2 are the characteristic isotropic g-values for the two spin species (in the present case $g_{1,2}$ = 1.998, the average g-value of Zn^I) and β_e is the Bohr magneton. As an example assuming a random distribution (spherical average), to inter-spin distances of 2 and 5 nm correspond dipolar frequencies of 6.4771 and 0.4145 MHz, respectively. With a magnetic dipole moment 660 times larger than that of a proton (~-9.285x10^{-24} JT^{-1} as opposed to ~1.411x10^{-26} JT^{-1}), an electron spin gives access to distances up to ≈ 15 nm, which are much longer than those accessible through NMR.

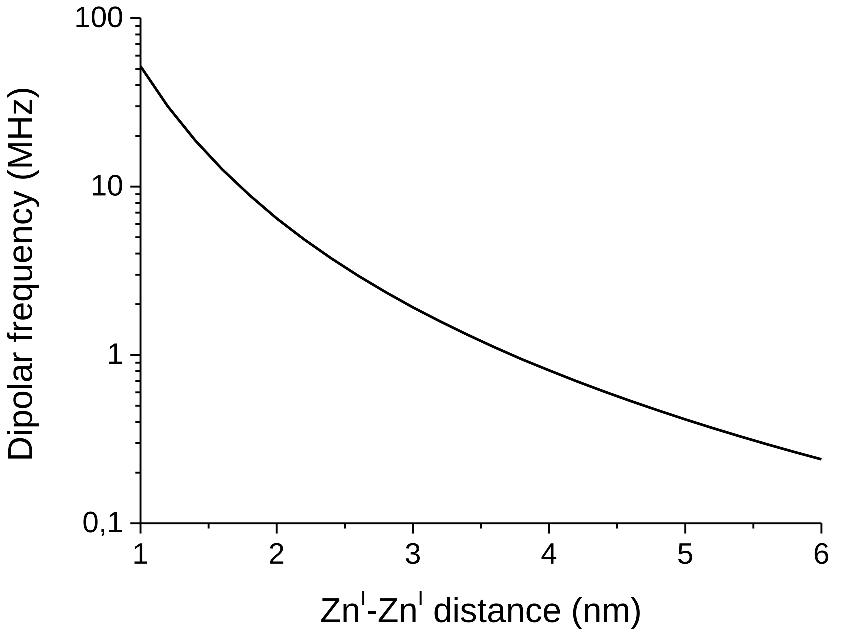

Fig. S3: Dependence of the dipolar frequency on the Zn^I-Zn^I interspin distance in the range 1 to 6 nm. The dipolar frequencies were calculated for a spherical distribution of spins according to the equation $\omega_{dipolar} = \frac{\mu_0 \beta_e^2}{4\pi\hbar} \frac{g_1 g_2}{r^3}$ and assuming g_1=g_2=1.998. The y-axis is reported in log-scale.

THE RIDME PULSE SEQUENCE

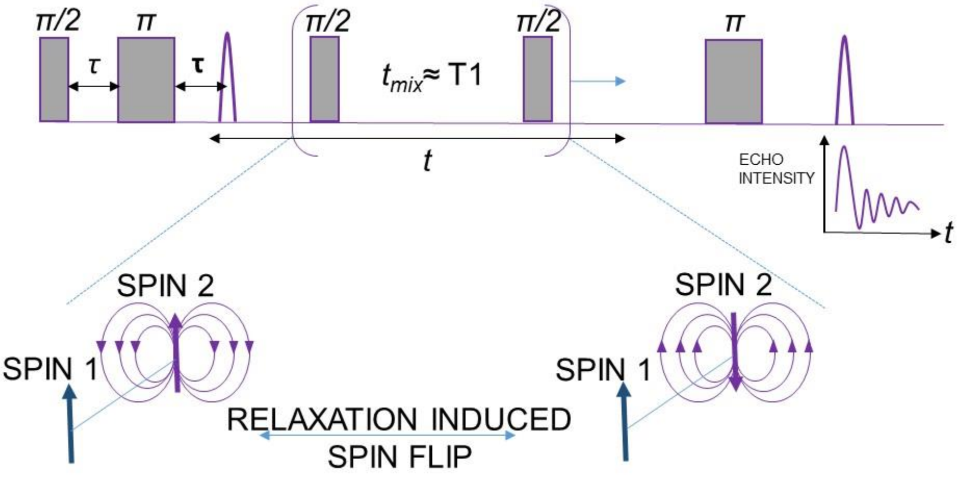

Fig. S4: Schematic representation of the RIDME pulse sequence and the physical process responsible for the observed dipolar modulation(s).

To understand the RIDME [3] sequence it is convenient to divide the spins in the samples into two groups: spins A are those excited by the microwave pulses at the working magnetic field; spins B are all other spins especially those dipolar coupled to spins A. Hence, in the sequence the first $\pi/2$ pulse generates electron coherence on A, which is then refocused by the first π pulse to generate a primary echo. From this time point, the electron coherence defocuses again until the second $\pi/2$ pulse transfers half of it along the z direction; this has the effect of forming a non-equilibrium magnetization grid for A along the direction of the external magnetic field. The grid is stored during the mixing time t_{mix} during which T_1 drives spontaneous spin flips of B, changing the magnetic dipole interaction and the resonance frequency of A. For RIDME efficiency (i.e. deep modulation depth), it is of paramount importance that spin B flips an odd number of times during t_{mix}. The third $\pi/2$ pulse has the effect of transferring back the A magnetisation onto the transverse plane from where it is refocused by the last π pulse to generate the RIDME echo. The RIDME time trace is a measure of the amplitude of the RIDME echo as a function of the position of mixing block ($\pi/2$-t_{mix}-$\pi/2$) in the time window between the two π pulses. Given that RIDME relies on spontaneous relaxation events, it is fundamental that t_{mix} is of the order of T_1. Moreover, being a single frequency experiment, RIDME can be performed using a resonator with a narrow bandwidth (high Q-factor). Moreover, RIDME is generally more sensitive than other PDS techniques, e.g. DEER/PELDOR, as it is not restricted by the bandwidth of the pump pulse.

The longest distance measurable is determined by the length of the dipolar trace, which in turn is determined by the relaxation times. Decreasing the working temperature, hence increasing the relaxation times, comes at the cost of speed of measurement. Therefore, a compromise must be sought between accessible distances and sensitivity.

RIDME PRIMARY DATA

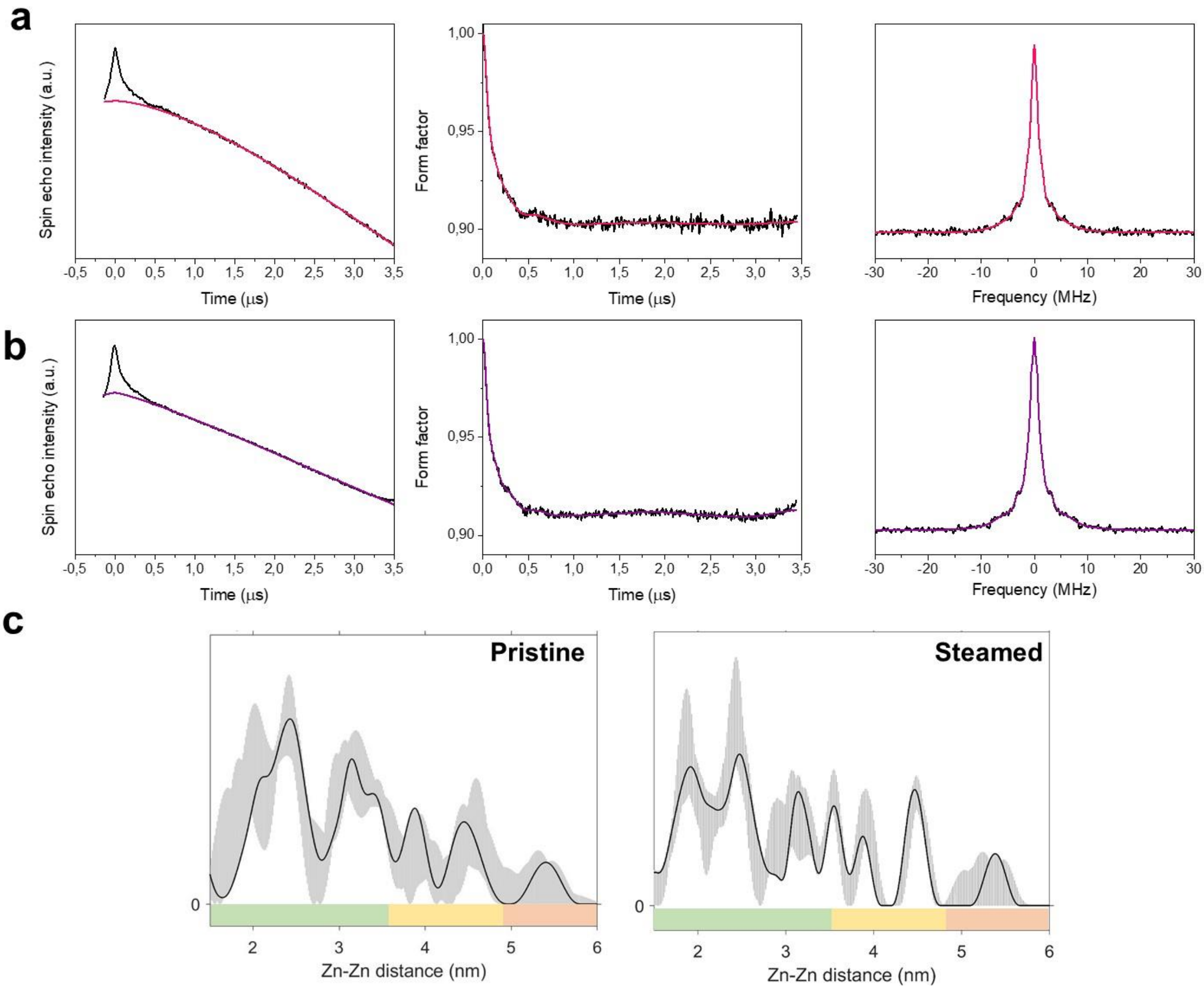

Fig. S5: **a**, RIDME primary data for the pristine ZSM-5 sample (black) with stretched exponential background (red), experimental form factor (black) and corresponding fitting (red) obtained through DeerAnalysis and Fourier transform of the form factors (experimental, black and simulated, red). **b**, RIDME primary data for the steamed ZSM-5 sample (black) with stretched exponential background (purple), experimental form factor (black) and corresponding fitting (purple) obtained through DeerAnalysis and Fourier transform of the form factors (experimental, black and simulated, purple). **c**, Validation analysis on the distance distribution obtained through DeerAnalysis. the solid lines represent the distributions with the best r.m.s. deviation, and the shaded regions represent the variation of alternative distributions obtained by varying the parameters of the background correction and noise. This analysis proves that the observed shift at low distances is robust against changes in the fitting parameters. The coloured bars on each panel represent the reliability of each region of the distribution, which is dependent on the length of the RIDME time trace, as defined in DeerAnalysis [4] (green: the shape of the distance distribution is reliable; yellow: the mean distance and distribution width are reliable; orange: only the mean distance is reliable).

RIDME DATA ON INDEPENDENT SAMPLE PREPARATIONS

To prove the reliability of the method, we performed RIDME measurements on an independent preparation of the Zn-ZSM-5 sample (Si/Al = 40, the same reported in the main text) and on a sample affording Si/Al = 15. As a consequence of the procedure used to introduce the Zn^{I} into the zeolite framework - direct sublimation of metallic zinc onto an activated zeolite - it is not always possible to achieve the same labelling efficiency, which in turn determines the attainable signal-to-noise ratio. The RIDME experimental data and corresponding fitting obtained through DeerAnalysis are reported in Fig. S6.

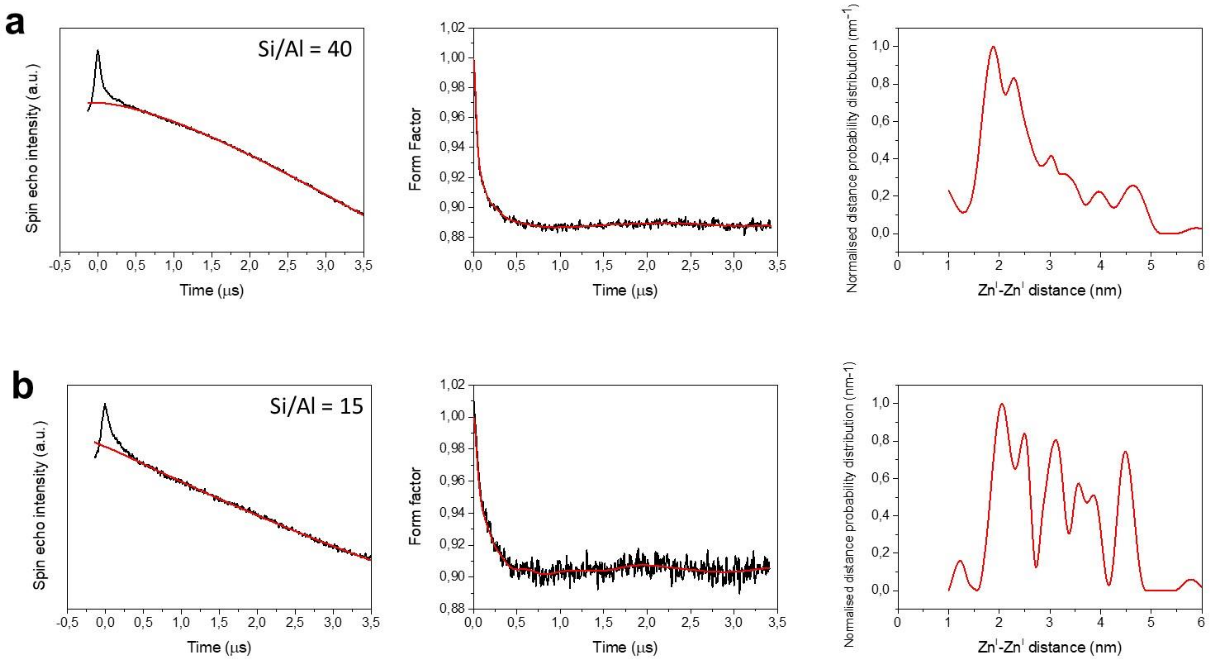

Fig. S6: **a**, RIDME data for a ZSM-5 Si/Al = 40 sample prepared independently from the sample presented in the main text (black) with stretched-exponential background (red), experimental form factor (black) and corresponding fitting (red) obtained through DeerAnalysis, corresponding distance distribution. **b**, RIDME data for ZSM-5 Si/Al = 15 sample (black) with stretched-exponential (red), experimental form factor (black) and corresponding fitting (red) obtained through DeerAnalysis, corresponding distance distribution.

The comparison between the Zn^{I}-Zn^{I} distances derived from the analysis of the form factors shows that the relative amplitude of the distance distribution peaks varies from one sample to the next. This is to be expected because in labelling inorganic crystalline structures with paramagnetic tags it is not possible to follow the same rules normally employed for biological samples. Indeed, it is in general impossible to achieve 100% occupancy and a large number of distances is in principle present due to multiple anchoring sites and the repetitive nature of the structure. Importantly, the peaks between 1.5 and 3 nm are present in each sample, proving that their origin real and reflect a structural feature of the material.

MONTE CARLO COMPUTATIONS ON ALL T SITES

The ZSM-5 zeolite belongs to the MFI topology and to the pentasil family since the 3D structure is built upon pentasil units each composed of eight five-membered rings. Oxygen bridges link the pentasil units together to yield pentasil chains which in turn generate a complex tridimensional architecture of cavities and channels. The latter can be classified into two classes: straight and sinusoidal and at their intersection they form large cavities. The space group of ZSM-5 is temperature dependent being orthorhombic (T > 300-350 K) or monoclinic (T < 300-350 K). The former is the high symmetry phase with only 12 independent T sites (T1 to T12), the latter is the low symmetry phase with 24 independent T sites (T1 to T24). Therefore, we did not limit our analysis to the T sites previously identified as most likely anchoring sites for Zn^{I}, but we extended it to all possible sites. The outcome of the computations is reported in Fig. S7.

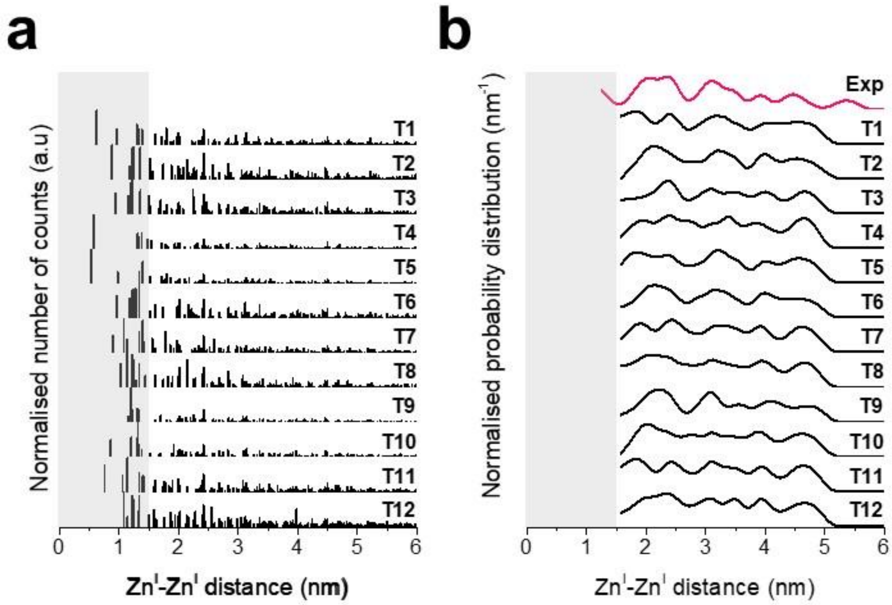

Fig. S7: **a**, Histograms reporting the normalised distribution of the $Zn^{I}\cdots Zn^{I}$ distances in the range 0 to 6 nm as computed through the Monte Carlo approach for each individual site from T1 to T12. Each distance distribution has a resolution of 0.02 nm and is the result of 20000 replicas. **b**, Model-free distance distributions (computed through DeerAnalysis, black lines) compared with the experimentally obtained distance distribution (red line). In both panels only the sites from T1 to T12 have been considered. The remaining sites (T13 to T24) display only minor differences as compared to the homologous ones (T1→T13, T2→T14 and so forth) and are therefore omitted.

THE ESEEM EFFECT

A known issue related to the RIDME pulse sequence is the enhancement of the so called ESEEM (Electron Spin Echo Envelope Modulation) effect, which stem from the hyperfine coupling between the electron spin and the surrounding nuclear spins. This may prove problematic, as hyperfine modulation may be mistakenly interpreted as dipolar frequencies and hence distances [5]. As it can be seen in the CW spectrum reported in Fig. S2, the Zn^{I} electron spin is hyperfine coupled to a ^{27}Al (I = 5/2). In previous studies we have fully established experimentally [1] and through DFT computations [2] the isotropic hyperfine coupling (a_{iso}), the dipolar hyperfine coupling tensor (T) and the nuclear quadrupole tensor (e^2qQ/h and η). Therefore, we can assess whether and how ESEEM would affect the measure distance distribution. In order to do that we simulated the 2p-ESEEM for a Zn^{I} spin coupled to ^{27}Al and run the simulated trace through DeerAnalysis [4]. As reported in Fig. S8, DeerAnalysis interprets the (fast) hyperfine modulations are as distances (main peak at 1.50 nm, lower contribution at ≈ 4.5 nm), although the fitting is rather poor. To account for this possible interference, in our analysis, we employed a cut-off filter at 1.6 nm.

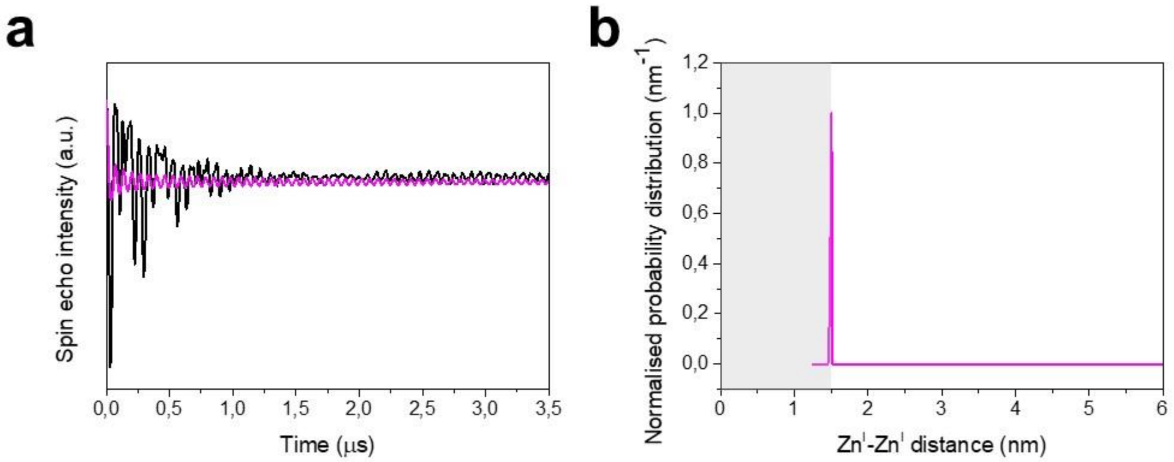

Fig. S8: **a**, Simulated 2-pulse ESEEM time trace (black) and corresponding fitting (magenta) obtained through DeerAnalysis. **b**, Distance distribution as obtained with DeerAnalysis. Hyperfine and nuclear quadrupole parameters considered: a_{iso} = + 3.36 MHz, T = [-0.56, -0.66, +1.22] MHz, e^2qQ/h = 9 MHz, η = 1 [1], initial interpulse delay τ = 200 ns, time increment 8 ns, magnetic field 1207 mT, microwave frequency = 33.7658 GHz, microwave excitation bandwidth 31 MHz. A noise level of 50 was added to make the simulations comparable with the experimental data.

DEER SPECTROSCOPY

Double electron-electron resonance (DEER) spectroscopy (also known as PELDOR, pulsed electron double resonance) is a PDS technique to measure distances between pairs of spins. Similar to RIDME, DEER measures an oscillatory time-domain signal that depends on the magnetic dipole–dipole coupling between the spins. However, it achieves this by employing two distinct microwave frequencies in a pump-probe experiment rather than relying on relaxation-induced spin flips, as illustrated in Fig. S9. The inversion of spin 2 by the pump pulse (mw2) leads to a change in the local magnetic field at spin 1 due to the dipolar coupling, $\omega_{dipolar}$.

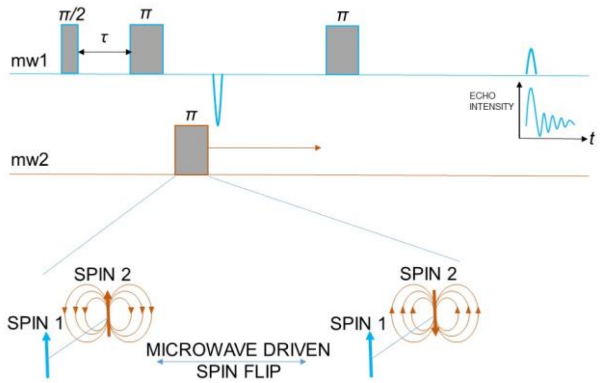

Fig. S9: Four-pulse DEER/PELDOR pulse sequence and the physical process responsible for the observed dipolar modulation(s).

As a point of comparison, 4-pulse DEER measurements were recorded on the pristine Zn-ZSM-5 zeolite at room temperature, placing the pump and the probe frequencies on the high- and low-field shoulders of the EPR spectrum respectively. The DEER data reported in Fig. S10 are the result of an eight-hour measurement. Despite the unsatisfactory signal-to-noise, we attempted an analysis employing the DeerAnalysis software. 4p-DEER confirms that both long and short distances are present, but appears to underestimate the contribution of the short distances, possibly lost in the noise. We suggest that the poor signal-to-noise stems from the narrow width of the EPR spectrum and the need of using relatively long and selective microwave pulses to avoid spectral overlap between pump and probe frequencies.

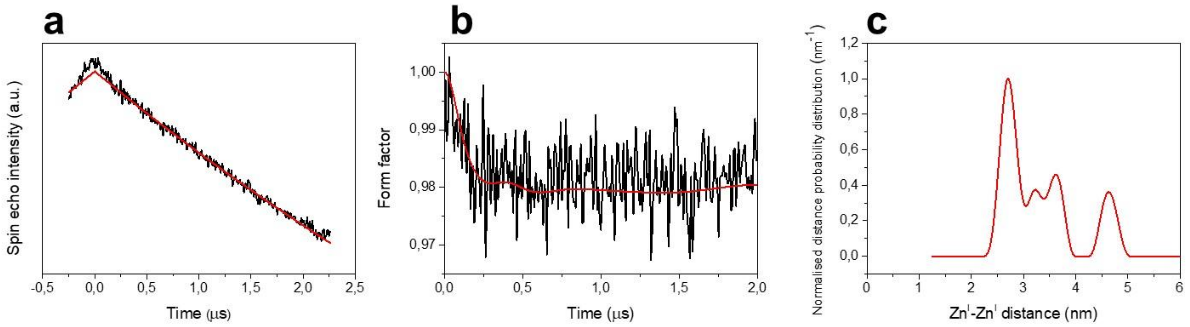

Fig. S10: **a**, DEER primary data for the pristine ZSM-5 sample (black) with homogeneous three-dimensional exponential (red). **b**, experimental form factor (black) and corresponding fitting (red) obtained through DeerAnalysis. **c**, corresponding distance distribution.

THE INDEPENDENT SITES APPROXIMATION

In principle, if a spin (termed A) is coupled to more than one other spin (termed for instance B and C) the dipolar trace is the product of each individual dipolar coupling (in the example AB x AC). However, if in a sample not all spins A are coupled to both B and C at the same time but some are coupled to B while others are coupled to C, then the dipolar trace is the sum of each individual component (in the example AB + AC). Fig. S11 schematically illustrates this idea. Given the low Zn^{I} occupancy, we made use of this approximation in computing the dipolar trace shown in the main text.

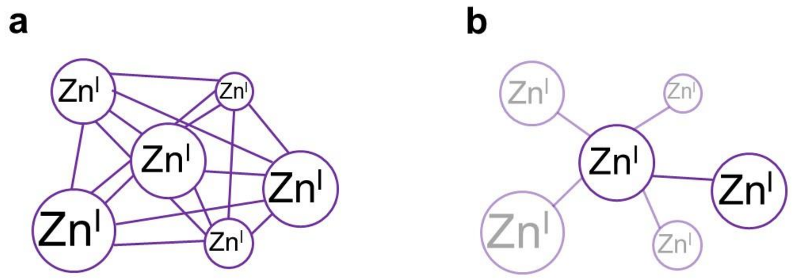

Fig. S11: **a**, Schematic representation of all possible dipolar coupling in a system composed of 5 Zn^{+} ions. **b**, Schematic representation for a sparsely populated system when only on Zn^{I}-Zn^{I} pair is present.

EFFECT OF THE Zn^{I} OCCUPANCY

To assess whether the percentage of Zn occupancy affected the calculated distance distribution, a number of trials at different occupancy levels (namely 0.1%, 1% and 5%) where conducted. Fig. S12 shows that the distance distribution in all cases is virtually identical. This is a consequence of the crystalline and ordered nature of the framework and the large number (20000 for 0.1 and 1 % and 10000 for 5%) of independent replicas considered.

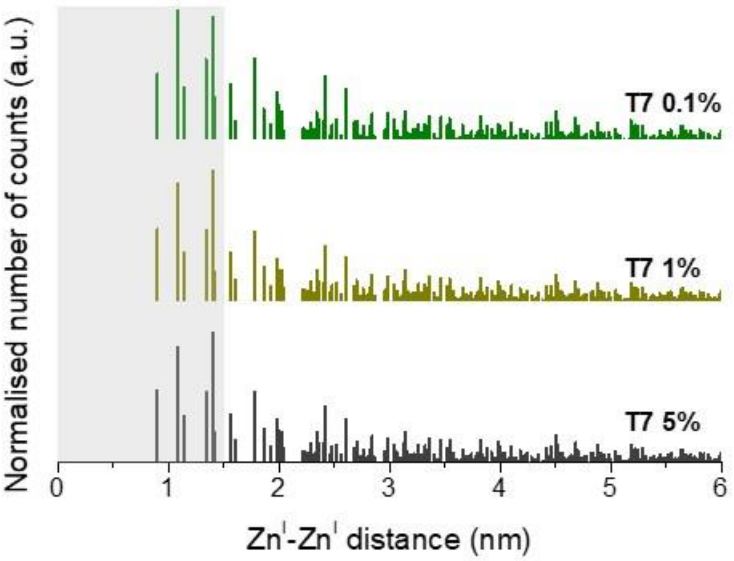

Fig. S12: Comparison of the for three loading of Zn^{I}, namely 0.1, 1 and 5 %. The area shaded in grey was not considered for the computation of the form factor.

REFERENCES

[1] Morra, E., Berlier, G., Borfecchia, E., Bordiga, S., Beato, P., Chiesa, M. Electronic and Geometrical Structure of Zn+ Ions Stabilized in the Porous Structure of Zn-Loaded Zeolite H-ZSM-5: A Multifrequency CW and Pulse EPR Study. *Journal of Physical Chemistry C* **121(26)**, 14238-14245 (2017)

[2] Morra, E., Signorile, M., Salvadori, E., Bordiga, S., Giamello, E., Chiesa, M. Nature and Topology of Metal–Oxygen Binding Sites in Zeolite Materials: ^{17}O High-Resolution EPR Spectroscopy of Metal-Loaded ZSM-5. *Angew. Chem. Int. Ed.* ***58***, 12398 (2019).

[3] Milikisyants, S., Scarpelli, F., Finiguerra, M.G., Ubbink, M., Huber, M. A pulsed EPR method to determine distances between paramagnetic centers with strong spectral anisotropy and radicals: the dead-time free RIDME sequence. *J. Magn. Res*. **201**(1), 48-56 (2009).

[4] Jeschke, G., Chechik, V., Ionita, P., Godt, A., Zimmermann, H., Banham, J., Timmel, C.R, Hilger, F., Jung, H. DeerAnalysis2006 — a comprehensive software package for analyzing pulsed ELDOR data. *App. Magn. Res*. **30**, 473-498 (2006).

[5] Keller, K., Doll, A., Qi M., Godt, A., Jeschke, G., Yulikov, M. Averaging of nuclear modulation artefacts in RIDME experiments. *Journal of Magnetic Resonance* **272**, 108-113 (2016).
